# Innate Immune Function of Mitochondrial Metabolism

**DOI:** 10.3389/fimmu.2017.00527

**Published:** 2017-05-08

**Authors:** David Sancho, Michel Enamorado, Johan Garaude

**Affiliations:** ^1^Centro Nacional de Investigaciones Cardiovasculares Carlos III (CNIC), Madrid, Spain; ^2^Institute for Regenerative Medicine and Biotherapy, INSERM U1183, Montpellier, France

**Keywords:** innate immune response, immunometabolism, electron transport chain, mitochondria, macrophages, dendritic cells, cytokines, inflammation

## Abstract

Sensing of microbe-associated molecular patterns or danger signals by innate immune receptors drives a complex exchange of information. Innate receptor signaling not only triggers transcriptional events but also induces profound changes in metabolic fluxes, redox balance, and metabolite abundance thereby influencing immune cell function. Mitochondria are at the core of metabolic adaptation to the changing environment. The close interaction between mitochondrial metabolism and immune signaling has emerged as a central regulator of innate sensing. Metabolic processes generate a constant flow of electrons that eventually end up in the mitochondrial electron transport chain (ETC). Two electron carriers and four respiratory complexes that can assemble as larger molecular supercomplexes compose the ETC in the mitochondrial inner membrane. While the meaning and biological relevance of such structural organization is a matter of passionate debates, recent data support that innate stimuli remodel the ETC. We will review the function of mitochondrial metabolism and ETC dynamics as innate rheostats that regulate signaling, transcription, and epigenetics to orchestrate innate immune responses.

## Introduction: Myeloid Cells Reprogram Their Metabolism in Response to Environmental Cues

Ligation of pattern recognition receptors, cytokine receptors, and phagocytosis of dying or dead cells provoke key changes in myeloid cell metabolism that are only beginning to be explored (1). As an example, tissue damage-derived signals can modulate myeloid cell metabolic reprogramming since uptake of apoptotic cells increases the mitochondrial membrane potential to downregulate phagocytosis (2). Another relevant example of metabolic consequences induced by innate immune signal is the stimulation of mouse macrophages and bone marrow-derived dendritic cells differentiated with GM-CSF (GM-DCs) with agonists for toll-like receptors (TLRs) or for the β-glucan receptor Dectin-1, which result in aerobic glycolysis (the Warburg effect) (1, 3–8). Many recent reviews have already remarkably described the recent advances in our understanding of metabolic reprogramming from a “metabolic flux” point of view (1, 9–13). However, recent works have provided new elements on the mechanisms bridging innate immune recognition and mitochondrial metabolic functions, suggesting that mitochondria regulate their electron flow as adaptation to innate immune signals. Here, we will summarize some of these findings that point toward a key function of the mitochondrial respiratory chain in governing innate immune cell fate.

## Mitochondria, a Metabolic Rheostat for Innate Immune Receptors

### Metabolism As a Flow of Electrons

Metabolism is often seen as a complex arrangement of metabolic fluxes, redox signaling, and translational or posttranslational events that are mutually dependent. However, metabolism can also be seen as a flow of electrons through multiple parallel and alternative pathways where metabolites act as potential carriers of electrons. From this point of view, the main and ultimate acceptor of electrons is the mitochondrial electron transport chain (ETC). Many catabolic processes indeed supply electrons to the ETC in the form of reducing equivalents of nicotinamide adenine dinucleotide (NADH) or flavin adenine dinucleotide (FADH_2_), whose relative proportion depends on the nature of the fuel used (14). The capacity of the cell to use different fuels efficiently is thus critical for its ability to adapt to changing environmental cues (15). Mitochondria must therefore regulate their location, biogenesis, fusion or fission, structure, and internal metabolite fluxes in response to changes in fuel source or signals received by cell membrane receptors or intracellular sensors. In turn, mitochondria control cell metabolism by governing the balance of anabolism (lipogenesis and antioxidant defenses from citrate, gluconeogenesis, serine, and glycine biosynthesis from pyruvate, nucleotide biosynthesis) and catabolism (Krebs cycle, β-oxidation, oxidative phosphorylation) (16). Mitochondria are central for ATP synthesis, redox balance, reactive oxygen species (ROS) production, thermogenesis, and generation of metabolites, all of which impact cell function. Since the release of cytochrome *c* to the cytosol is a major trigger for apoptosis, mitochondria also regulate cell survival.

**Figure 1:**
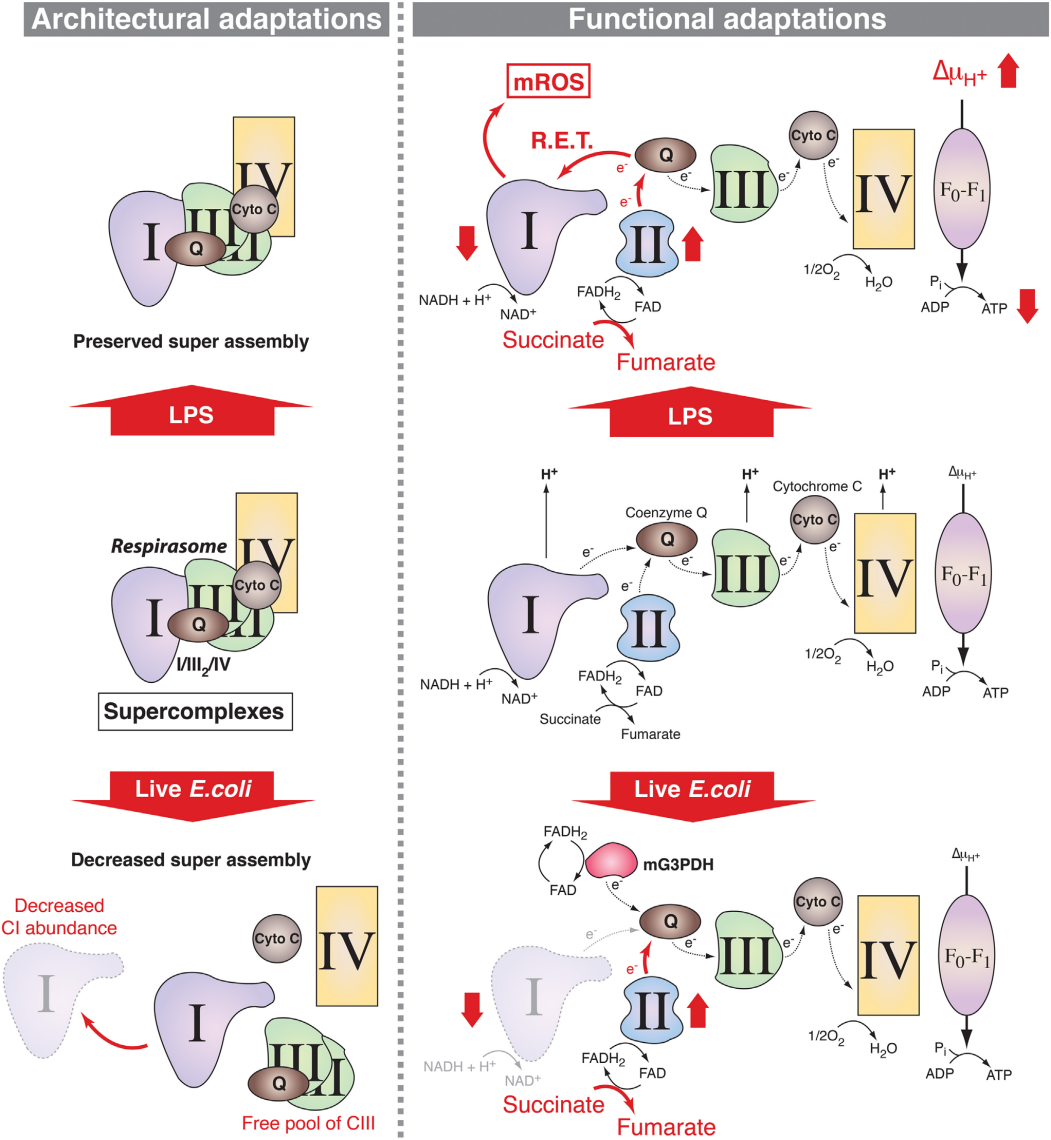
**Mitochondrial respiratory chain adaptations following innate immune sensing**. Mitochondrial respiratory complexes, except for CII, can associate into supercomplexes (SCs) including the respirasome, composed of CI + CIII_2_ + CIV (left panel). In lipopolysaccharide (LPS)-stimulated mouse macrophages, SC assembly is preserved (upper left) but there are functional adaptations such as increase in CII (succinate dehydrogenase) activity, which enhances succinate oxidation along with increased mitochondrial membrane potential and decreased mitochondrial ATP synthase-mediated production of ATP (upper right). This is accompanied by a decrease in the NAD^+^/NADH ratio, supporting a possible reverse electron transport (RET) from coenzyme Q to CI thereby inducing the production of mitochondrial reactive oxygen species (mROS). Upon detection of live *Escherichia coli*, increase in phagosomal reactive oxygen species mediates Fgr-dependent activation of CII and decrease in CI-containing SCs probably due to CI disassembly. This provokes a change in electron flow in the electron transport chain, with a drop in the entry of electron derived from NADH that is compensated by the induction of the activities of FADH_2_-consuming enzymes CII and mitochondrial glycerol-3-phosphate dehydrogenase (mG3PDH). The increase in CII activity generates fumarate that modulates macrophage function.

The ETC comprises two electron carriers [coenzyme Q (CoQ)/ubiquinone and cytochrome *c*] and four respiratory complexes [complexes I–IV (CI–CIV)], which, excluding CII, can assemble as larger molecular supercomplexes (SCs) in the mitochondrial inner membrane (16) (Figure 1). The assembly of SCs is dynamic and may adapt the electron flux to the available substrates (17, 18). The nature of the fuel conditions the proportion of electrons feeding into the ETC from NADH and FADH_2_, with a NADH/FADH_2_ electron ratio of 5 following full oxidation of glucose and a ratio of 2 following oxidation of the fatty acid palmitate (14, 17, 18). The H^+^ gradient generated by the ETC is then used by the H^+^-ATP synthase (CV) to generate ATP. Recent results support the notion that innate sensing of microbial features leads to adaptations in SC assembly and electron flow through the ETC in mouse macrophages (5, 19) suggesting that ETC exerts key functions in macrophage activation processes (Figure 1).

### Mitochondria As Platforms for Innate Signaling

Mitochondria can both generate ligands and serve as signaling platform for innate sensing receptors (12). First, some mitochondrial-derived molecules trigger immune receptors. This is the case for mitochondrial *N*-formyl peptides, which are damage-associated molecular patterns that activate receptors such as formyl peptide receptor-1 to promote cytokine production (20). Furthermore, mitochondrial DNA (mtDNA) has hypomethylated CpG that can trigger TLR9 activation (20). mtDNA can access the cytosol through an altered permeability of the mitochondrial membrane to activate the NLRP3 inflammasome (21, 22). Later, it was discovered that glycolytic enzymes can directly contribute to innate sensing of microbes. Hexokinase is a glycolysis enzyme associated with the voltage-dependent anion channel (VDAC) in the outer mitochondrial membrane (23). Following degradation of microbe-associated peptidoglycans in the phagosomes of mouse macrophages and DCs, peptidoglycan-derived *N*-acetylglucosamine binds to hexokinase causing its dissociation from the mitochondria outer membranes and VDAC (24). The NLRP3 inflammasome is subsequently activated, possibly relying on variations of the mitochondrial membrane permeability and the access of mtDNA to the cytosol. Thus, hexokinase moonlights as a regulator of NLRP3 activation and subsequent maturation of pro-IL-1β to promote an antibacterial pro-inflammatory response (24, 25). In addition, cytosolic location of mtDNA can also induce antiviral immunity by triggering the DNA sensor cGAS and the STING-IRF3-dependent pathway to promote IFN-I production (26).

Another example of how the outer mitochondrial membrane acts as a scaffold structure involved in innate sensing is the mitochondrial antiviral-signaling protein (MAVS) that binds to mitochondrial-associated membranes connecting the endoplasmic reticulum to the outer mitochondrial membrane (27). RIG-I binds to and promotes MAVS aggregates in the outer mitochondrial membrane to trigger downstream signaling (28). Thus, signaling and adaptor proteins downstream of pattern recognition receptor (PRR) sensing pathways can localize to mitochondria raising the possibility that PRRs could modulate mitochondrial functions. Such a regulatory role has been already suggested for tumor necrosis factor receptor-associated factor 6 (TRAF6), which can interact with evolutionarily conserved signaling intermediate in toll pathways (ECSIT), a protein involved in CI assembly (29, 30).

### Metabolic Reprogramming in Macrophages upon Innate Immune Receptor Engagement

The specificities of macrophage metabolism compared to other immune cells have been a subject of interest for a long time as exemplified by pioneer works from the 80s (31–33). Our current appreciation of metabolic reprogramming led us to postulate that most inflammatory agonists for PRRs engage similar metabolic adaptations, although some specificities on the outcomes for host defense may exist. The main characteristic of metabolic adaptations upon innate immune receptor engagement is a strong induction of glycolysis even in presence of substantial oxygen (1, 3–8). Indeed, mouse macrophage stimulation with the TLR4 agonist lipopolysaccharide (LPS), the main component of Gram-negative bacterial cell wall, induces the activation of transcription factor hypoxia-inducible factor-1α (HIF-1α) (8) that controls the expression of several enzymes implicated in glycolysis (34, 35). LPS-activated mouse macrophages express a highly active isoform of phosphofructokinase-2 that promotes glycolysis (36) and LPS induces pyruvate kinase M2, which associates with and stabilizes HIF-1α to further induce glycolysis and enhance proinflammatory cytokines (37, 38). Such induction of glycolytic flux was also found in phagocytic cells activated through TLR2 (4, 39), TLR3 (4, 5), TLR7/8 (4, 5), TLR9 (4, 40), or Dectin-1 (3, 41), indicating that enhanced glycolysis might be a common feature to PRR-activated cells. The detection of a number of microbes including *Salmonella typhimurium* (5, 37), *Escherichia coli* (5), or *Mycobacterium tuberculosis* (37, 42, 43) strongly induce glycolysis in mouse macrophages. However, inflammasome activation of caspase-1 mediates the cleavage of glycolytic enzymes, e.g., during NLRC4 sensing of *S. typhimurium* (44) highlighting the complex interplay between PRRs and myeloid cell metabolism. In fact, induction of glycolysis does not seem to solely contribute to metabolic reprogramming but might also directly sustain pathogen sensing and host defense.

Although glycolysis is thought to largely contribute to ATP production in activated myeloid cells (40, 45), it also provides metabolic intermediates that could feed other metabolic pathways to serve macromolecule synthesis (45). Along with glycolysis, the pentose phosphate pathway (PPP) is induced in LPS-activated mouse macrophages (7). The mechanisms engaged are not fully understood, but they likely involve the inhibition of the PPP inhibitor carbohydrate kinase-like protein (46).

Because the glucose catabolic product pyruvate is diverted from entry to mitochondria in activated macrophages and is rather metabolized to lactate, it was tempting to speculate that engagement of innate immune receptors could globally dampen mitochondrial respiration. However, innate stimulation also activates pathways such as glutaminolysis to replenish the tricarboxylic acid (TCA) cycle and maintain global metabolic flux (7, 8, 47) likely avoiding cell death. Thus, a significant activity of the mitochondrial respiratory chain and the mitochondrial membrane potential must be maintained. This is partially achieved by an increase in anaplerosis including glutaminolysis (7, 8), which feeds the TCA cycle at α-ketoglutarate, and the aspartate–argininosuccinate shunt, which feeds the TCA cycle at malate and fumarate (7). Glutaminolysis is also enhanced in human monocytes trained with β-glucan, which triggers the C-type lectin receptor Dectin-1 (41, 48), suggesting that induction of such metabolic feature is not limited to TLRs. Therefore, an important issue to address is whether different PRRs induce specific metabolic reprogramming signatures, which would allow specific manipulation of myeloid cell metabolism for therapy.

How innate immune receptors regulate lipid metabolism in macrophages is a field of active research. On the one hand, engagement of TLRs upon recognition of various bacteria enhances fatty acid uptake and incorporation to triglycerides for storage and simultaneously decrease fatty acid oxidation and lipolysis suggesting that lipids may serve other needs than energy production in activated mouse macrophages (6, 49, 50). In line with this, fatty acid catabolism is not induced in LPS-activated macrophages compared to alternatively activated macrophages (7). Consistently, mouse macrophage fatty acid synthase is induced during sepsis in a mechanism depending on the mitochondrial uncoupling protein 2 (UCP2) (51). On the other hand, fatty acid oxidation is increased in mouse and human macrophages primed in conditions that activate the NLRP3 inflammasome, a process that requires mitochondrial NADPH oxidase 4 (NOX4)-dependent ROS production (52). Therefore, it is clear that needs for lipid metabolism in activated macrophages is significantly increased but whether this fulfills mitochondrial energy production requirements or rather constitutes the primal building blocks for anabolism needs to be further investigated.

### Mitochondrial Respiratory Chain Adaptations in Macrophages upon Innate Immune Receptor Engagement

As stated above, most catabolic processes converge on the mitochondrial ETC by supplying electrons in the form of the reductive equivalents NADH and FADH_2_. The intramitochondrial NADH/FADH_2_ ratio depends on the nature of the fuels that feed the mitochondrial metabolism and the respiratory chain adapts to these fuel source fluctuations (16), particularly during PRR-mediated macrophage activation. Indeed, two recent studies provide evidence that changes in the ETC occur in activated mouse macrophages (5, 19). The phagocytosis of live Gram-negative bacteria by mouse macrophages trigger a profound change in ETC structural organization (5) (Figure 1). This is characterized by a decreased abundance of ETC SCs that contain CI and a relative increase in the free form of CIII and is accompanied by a substantial decrease in the activity of CI. By contrast, the activity of the glycerol 3-phosphate dehydrogenase and CII, two enzymes that use FADH_2_, are increased in response to live bacteria or to TLR-mediated sensing of bacterial RNA. CII activity is driven by the production of phagosomal ROS. This activates the ROS-dependent tyrosine kinase Fgr, which was previously found to phosphorylate CII (53). Consistently, CII was essential to mitochondrial respiration in bacteria-activated macrophages. This work thus suggests that adjustments of the organization of the ETC and of the activity of its components are required for metabolic reprogramming in macrophages. A study by O’Neill and colleagues has further precised the mechanism by which ETC integrates signals emerging from TLRs (19) (Figure 1). The combination of enhanced succinate dehydrogenase (SDH) activity and increased mitochondrial potential in LPS-activated mouse macrophages allows for the induction of mitochondrial ROS production at the level of CI thereby reprogramming mitochondria from ATP production to ROS production in order to establish an inflammatory state. This is consistent with a previous study showing that metformin-mediated inhibition of CI-dependent ROS production suppresses the inflammatory capacity of activated mouse macrophages (54). These data point out CI as a major regulator of inflammatory macrophages. This is backed up by genetical evidences demonstrating that mouse macrophages deficient for the CI subunit NDUFS4 exhibit an inflammatory phenotype (55). Interestingly, the deletion of TLR2/4 in NDUFS4-deficient mice attenuated the inflammatory phenotype (55), highlighting the close relationship between TLRs and the ETC. However, the contributions of mitochondrial respiratory chain to innate immune functions seems not limited to TLRs since the engagement of Dectin-1 in human monocytes induces accumulation of CII-product fumarate, which can induce an epigenetic program accounting for trained immunity by inhibiting KDM5 histone demethylases (41). Whether adaptations of the ETC reflect solely the metabolic fluctuations within activated macrophages or can be directly regulated by signals emerging from innate immune receptors remains to be clarified.

## Functional Consequences of Mitochondrial Adaptations in Macrophages

### Regulation of Macrophage Polarization and Cytokine Response

The finding that IL-4, a well-known inducer of alternatively activated mouse macrophages (M2), promotes a distinct metabolic reprogramming compared to pro-inflammatory (M1) mouse macrophage stimulating LPS/IFN-γ indicates that mitochondrial metabolism adjusts to the type of immune responses required to eliminate the threats encountered (1). M1 mouse macrophages produce high amount of pro-inflammatory cytokines and antimicrobial peptides and excel in phagocytosing and destroying microbes. Those macrophages exhibit a high glycolytic rate and present two breaks in the TCA cycle, one at isocitrate dehydrogenase, the enzyme that converts isocitrate to α-ketoglutarate, and another one at SDH that catalyzes the oxidation of succinate to fumarate (7). M2 mouse macrophages are essential at fighting against helminth infection, exert tissue repair functions, and conserve an intact TCA cycle (7). The specificities of metabolic fluxes in differentiated macrophages is a subject of intense research and has been reviewed elsewhere (1). However, whether mitochondrial ETC organization and its functional adjustments reflect the metabolic specificities of M1 and M2 macrophages is still poorly understood. A recent report nevertheless showed that LPS + IFN-γ treatment promotes NO that inhibits mitochondrial respiration thereby preventing the repolarization of M1 into M2 macrophages in mice and humans upon IL-4 treatment (56). In line with this, various reports suggest that manipulating the ETC modulates the balance between pro- and anti-inflammatory cytokine production. The use of the CI inhibitors metformin and rotenone decreased mitochondrial ROS production in LPS-activated mouse macrophages thereby reducing IL-1β production and boosting IL-10 production (54). In addition to their function as TLR7 agonists, imiquimod and CL097 also inhibited the quinone oxidoreductases NQO2 and CI, inducing ROS that contributed to NLRP3 activation (57). Conversely, the CII inhibitor dimethyl malonate enhanced the production of IL-1RA and IL-10 and promote an anti-inflammatory response by preventing mitochondrial ROS generation and succinate oxidation (19).

**Figure 2:**
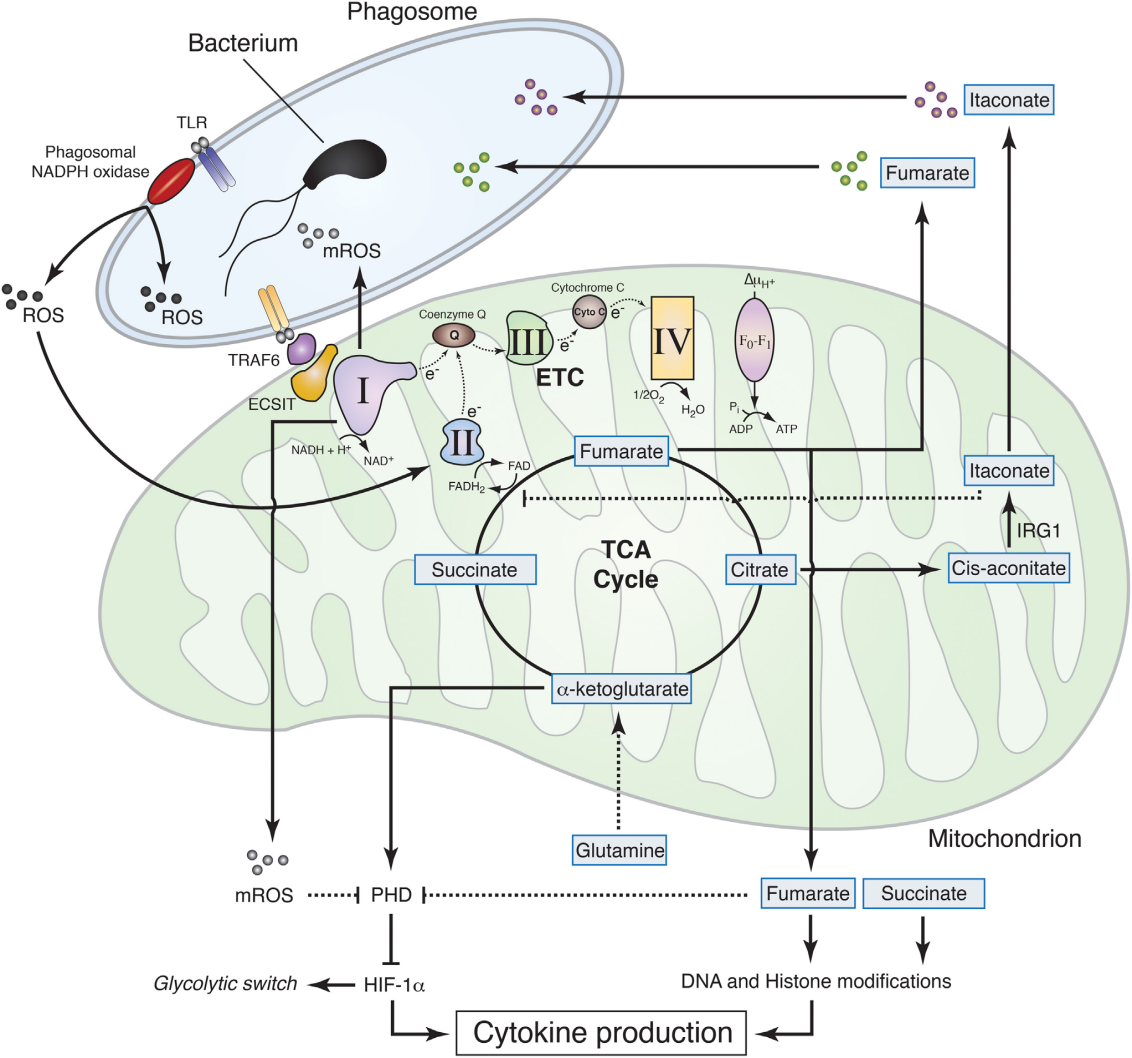
**Mitochondrial metabolism contributions to macrophage effector functions**. Sensing of live Gram-negative bacteria by toll-like receptors (TLR) induces the production of phagosomal reactive oxygen species (ROS) by phagosomal NADPH oxidase. Phagosomal ROS directly contribute to the killing of the bacteria inside the phagosome and induce CII activity thereby promoting fumarate accumulation, which exerts bactericidal properties and modulates histone posttranslational modifications to control cytokine production. The increase in CII activity is associated with a reduction in CI activity and a subsequent increase in mitochondrial ROS (mROS). TLR mediates recruitment of the mitochondria to the phagosome through tumor necrosis factor receptor-associated factor 6 (TRAF6)–evolutionarily conserved signaling intermediate in toll pathways (ECSIT) interaction and concomitantly increases mROS, which contribute to bacteria killing in the phagosome and inhibit prolyl hydroxylase (PHD), thus promoting hypoxia-inducible factor-1α (HIF-1α) stabilization. TLR signaling also induces accumulation of succinate, which, together with fumarate, inhibits α-ketoglutarate-dependent DNA hydroxylases and histone demethylases, regulating cytokine expression and glycolytic switch. Succinate oxidation, in turn, is required to induce mROS production at the level of CI, thereby inhibiting PHD, stabilizing HIF-1α, and further controlling cytokine production. In addition to fumarate, other tricarboxylic acid cycle-related metabolites exhibit antibactericidal properties. Citrate is used to produce itaconate through the decarboxylation of *cis*-aconitate by IRG1. Itaconate was found to have antimicrobial properties and to regulate succinate dehydrogenase (CII) activity in lipopolysaccharide-activated macrophages.

Mitochondrial metabolites, e.g., those related to α-ketoglutarate, such as succinate and fumarate, regulate transcription and influence the pattern of cytokine production that characterizes differentiated macrophages. Succinate is accumulated in M1 mouse macrophages and was originally thought to stabilize HIF-1α through inhibition of prolyl hydroxylases (58). However, new evidence shows that succinate rather drives CII activity, which in turn promotes mitochondrial reactive oxygen species (mROS) production to stabilize HIF-1α (19), thereby promoting pro-inflammatory cytokine IL-1β expression (8) (Figure 2). Succinate may also contribute to increased reverse electron transport and ROS production from CI (59), a process that is potentially important for macrophage function (60). Likewise, SDH (part of the CII) metabolizes succinate to fumarate in the TCA cycle, and fumarate inhibits histone demethylases thus regulating epigenetics (41). In this process, CII accepts the electrons from FADH_2_, and this function as electron carrier drives modulation of ROS signaling (60). These results establish how ROS drives expression of pro-inflammatory cytokines that characterize M1 macrophages but the mechanism by which succinate inhibits anti-inflammatory gene expression is still an open question. Taken together, those studies show the great potential of mitochondrial metabolism for regulation of transcription, cytokine production, and macrophage polarization that can be exploited for therapy.

### Regulation of Macrophage Bactericidal Functions

Although phagosomal ROS are considered to be a main component of bacteria killing machinery within macrophages, mitochondria seem to significantly contribute to antibacterial functions by generating a substantial amount of ROS (Figure 2). Engagement of a cell membrane-associated TLRs (TLR1, TLR2, and TLR4) results in the recruitment of mitochondria to macrophage phagosomes and increases mROS production. This response involves translocation of the TLR signaling adaptor TRAF6 to mitochondria where it engages the mitochondrial respiratory complex I assembly adaptor ECSIT (30). Consistently, mouse macrophages that lack ECSIT or TRAF6 show decreased levels of TLR-induced ROS and impaired ability to kill intracellular bacteria. In addition, reducing mouse macrophage mROS levels by expressing catalase in mitochondria results in defective bacterial killing, confirming the role of mROS in bactericidal activity (30). Additional studies support the notion that suppressing mROS production regulates antibacterial innate immune responses. Mice deficient for UCP2 and infected with a lethal load of *Toxoplasma gondii* were significantly more resistant compared with wild-type (WT) animals. Consistently, UCP2-deficient macrophages generated more ROS in response to *T. gondii* underlying this enhanced parasite-killing capacity (61). A subsequent study detailed a similar phenotype, demonstrating that *Ucp2^−/−^* mice were more resistant to *Listeria monocytogenes* and display higher splenic ROS levels than WT mice (62). LPS stimulation also reduces macrophage UCP2 expression, indicating that the abundance of UCP2 in the cell regulates mROS generation following TLR4 engagement (63). Additionally, it was demonstrated that IFNγ induces an estrogen-related receptor-α (ERRα)- and a proliferator-activated receptor-γ co-activator (PGC1β)-dependent transcriptional program in mouse macrophages that induces mitochondrial function and mROS production upon bacterial infection (64). Consequently, ERRα- or PGC1β-deficient macrophages produce less ROS upon *L. monocytogenes* challenge and display a reduced bacteria killing capacity (64). In this context, ETC re-arrangement may also regulate mROS production by controlling electron leak within the respiratory complexes (16). Future studies will likely provide additional details on the role of ETC architecture and electron flow for antimicrobial function of mitochondria.

The notion that mitochondria are important players for the microbicidal properties of macrophage has recently gained further interest with the finding that mitochondrial metabolites directly contribute to macrophage bactericidal functions. The mitochondrial protein immune responsive gene 1 (IRG1) is highly expressed in mouse and human macrophages during inflammation. It metabolizes *cis*-aconitate into itaconate, which can drive the production of β-oxidation-dependent mitochondrial ROS (65). Importantly, itaconate inhibits citrate-lyase in different bacterial strains and thus shows direct antimicrobial activity (66) (Figure 2). IFNs induce IRG1, which in turn accumulates in mitochondria that closely associate with *Legionella*-containing vacuoles, as step that may be required for bacteria killing (67). In addition, itaconate has been postulated to modulate mouse macrophage metabolism and effector functions by inhibiting SDH. Indeed, *Irg1^−/−^* mice, which cannot produce itaconate, have decreased level of succinate and increased mitochondrial respiration and inflammatory cytokine production upon LPS stimulation (68). Reorganization of the ETC may also participate to such processes, since the SDH product fumarate can directly inhibit bacteria proliferation in response to ETC adjustments upon sensing of viable bacteria (5) (Figure 2). Notably, the substrate of SDH succinate did not show antimicrobial properties as compared to fumarate, revealing specific immune properties of closely related mitochondria-derived metabolites (5).

## Concluding Remarks

Since mitochondria have emerged as key conductors of the metabolic adaptations to the innate sensing by modulating the electron flow in the ETC (5, 19), a key open question is whether targeting of mitochondrial metabolism can be used to modulate immune cell function with substantial improvement for therapeutic strategies. This would be particularly interesting for enhanced vaccination and modulation of immunity and tolerance when targeting DCs, or for the treatment of inflammatory diseases in the case of targeting macrophages. In the later, mitochondrial manipulation would constitute an approach of choice to facilitate macrophage repolarization (56). Because some mitochondrial metabolites and the use of ETC CI or CII inhibitors can modulate cytokine production (8, 19, 41, 54, 57), such approach seems very promising. Moreover, mitochondrial ROS and metabolites, such as fumarate or itaconate, show a direct microbicidal effect (5, 30, 66). The exciting findings in this rapidly moving field thus emphasize the great potential of targeting mitochondrial metabolism for modulation of transcription, polarization, cytokine production, and microbicidal capacity of macrophages with potential to offer new therapeutic approaches.

## Author Contributions

DS, ME, and JG conceived and wrote the manuscript. JG did the figures.

## Conflict of Interest Statement

The authors declare that the research was conducted in the absence of any commercial or financial relationships that could be construed as a potential conflict of interest.
